# 134. Impact of an Antibiotic Stewardship Treatment and Management Algorithm for Liver Abscesses

**DOI:** 10.1093/ofid/ofab466.336

**Published:** 2021-12-04

**Authors:** Hunter Vanderburg, Jacqueline Meredith, Rupal K Jaffa, Cesar Aviles, Benjamin M Motz, Allyson Cochran, Vivek Shastry, Dionisios Vrochides, Leigh Ann Medaris

**Affiliations:** 1 Atrium Health’s Carolinas Medical Center, Charlotte, North Carolina; 2 Atrium Health, Carolinas Medical Center, Charlotte, North Carolina; 3 Atrium Health, Charlotte, North Carolina; 4 Atrium Health, Carolinas Medical Center - Charlotte, Mount Holly, North Carolina; 5 Carolinas Medical Center - Atrium Health, Charlotte, North Carolina

## Abstract

**Background:**

Antibiotic prescribing for pyogenic liver abscess(es) (PLA) is highly variable with literature primarily aimed at assessing surgical intervention with a scarcity of data for antibiotic selection and duration of therapy. Given the lack of data, there is no clear consensus for treatment options or length of treatment. Our Antimicrobial Support Network (ASN) in collaboration with the hepatopancreatobiliary (HPB) team created a treatment and management algorithm to guide duration of therapy and antibiotic selection.

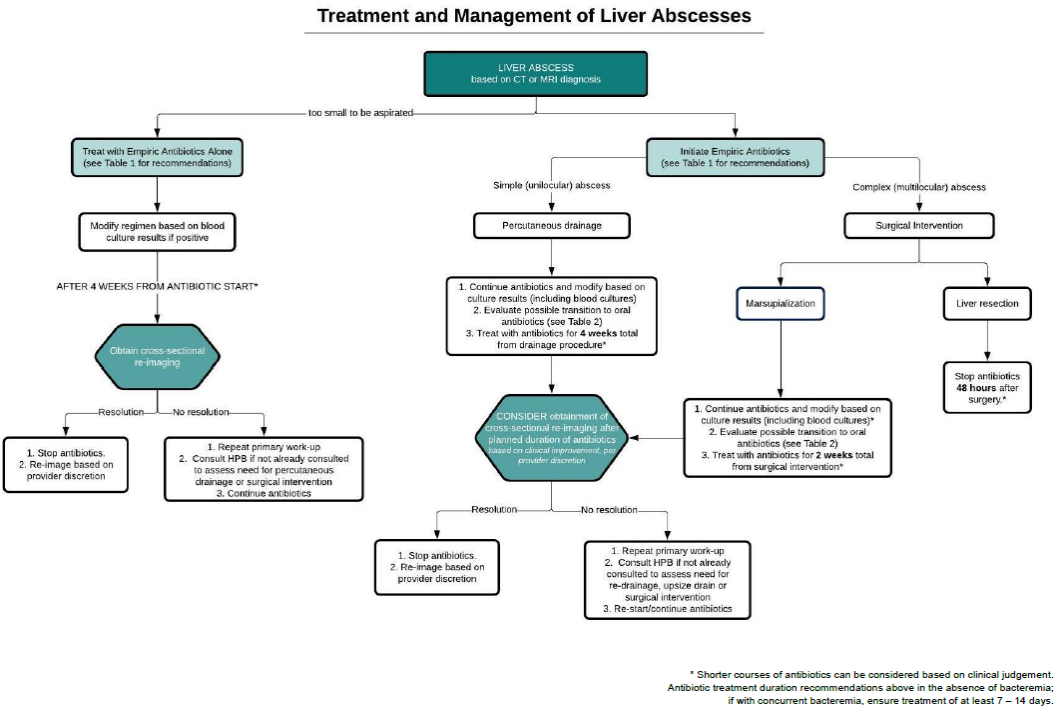

**Methods:**

A retrospective, quasi-experimental cohort study was performed at Carolinas Medical Center in hospitalized patients with PLA with an HPB and/or infectious diseases consult. The primary outcome was antipseudomonal beta-lactam days of therapy (DOT) per 1000 patient days (PD) in the pre-versus post-intervention group. Secondary outcomes included rates of treatment failure at 90 days, 90-day all-cause and abscess-related hospital readmission, *C. difficile* and multi-drug resistant organism (MDRO) colonization at 90 days from diagnosis, and hospital length of stay (LOS). Additional *a priori* subgroup analyses of duration of therapy, treatment failure, all-cause and abscess-related readmissions were also conducted based on surgical intervention.

**Results:**

A total of 93 patients were included, 49 patients in the pre-intervention group and 44 patients in the post-intervention group. Baseline characteristics were similar between the groups. The majority of liver abscesses were unilocular and monomicrobial. Anti-pseudomonal beta-lactam DOT per 1000 PD decreased by 13.8% (507.4 versus 437.5 DOT/1000 PD). Treatment failure occurred in 30.6% of pre-intervention patients and 18.2% of post-intervention patients (p = 0.165). Patients in the post-intervention group were discharged a median of 2.4 days sooner than the pre-intervention period (12.2 days vs. 9.8 days, p = 0.159). No significant differences resulted in 90-day readmission rates or 90-day *C. difficile* or MDRO rates.

Table 1. Primary Outcome for Patients with Pyogenic Liver Abscesses Treated Pre- and Post-Antibiotic Stewardship Algorithm

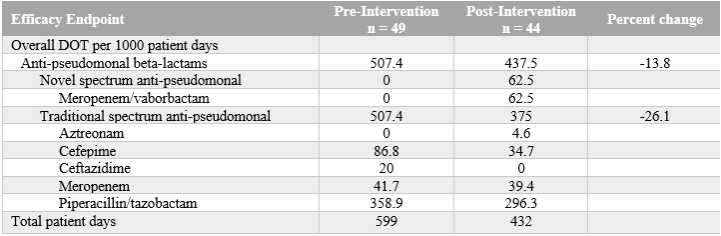

Table 2. Secondary Outcomes for Patients with Pyogenic Liver Abscesses Treated Pre- and Post-Antibiotic Stewardship Algorithm

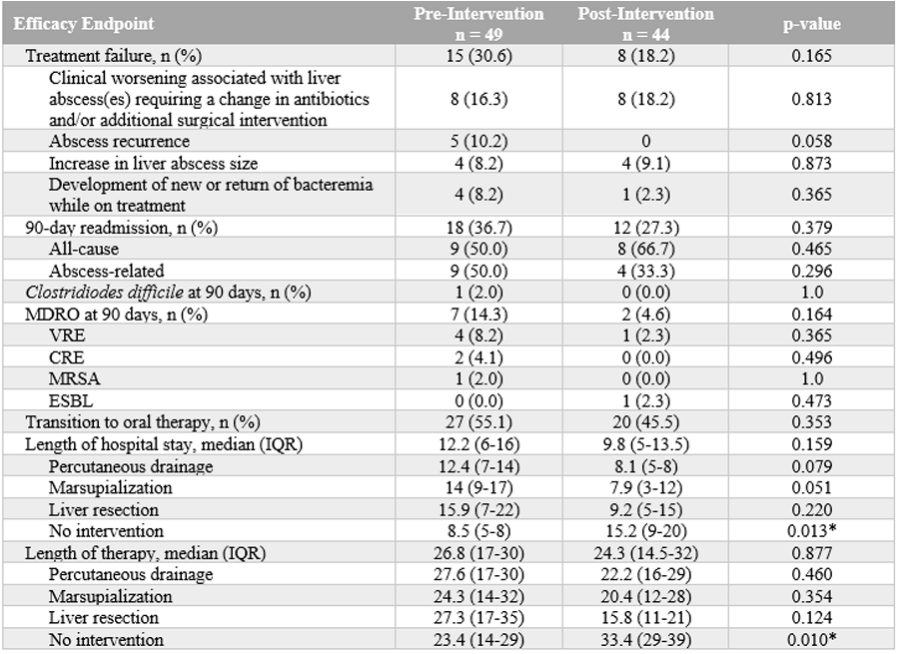

**Conclusion:**

The implementation of a PLA treatment and management algorithm led to a decrease in anti-pseudomonal beta-lactams without impacting clinical outcomes and a trend towards decreased LOS.

**Disclosures:**

**All Authors**: No reported disclosures

